# Stress distribution and deflection of symmetric tapered beams

**DOI:** 10.1007/s00707-025-04243-7

**Published:** 2025-02-12

**Authors:** Juergen Schoeftner

**Affiliations:** Institute of Technical Mechanics, Johannes Kepler University Linz, Altenberger Strasse 69, 4040 Linz, Upper Austria Austria

## Abstract

This paper discusses stress distributions and deflections of tapered beams. Assuming the elementary axial stress distribution and applying Jourawski theory, the shear stress is calculated. In an analogous manner, the transverse normal stress is computed. In contrast to prismatic beams, the shear and the transverse normal stress do not vanish over the surfaces if the beam height varies, even if surface tractions are not present. Their values also depend on the tapering angle and the axial stress at the boundary. Then analytical expressions for the deflections are computed by applying Castigliano’s second theorem and considering a fictitious (dummy) force. The complementary strain energy is computed from the derived stress relations as a function of the real load and the dummy forces and moments. Taking the partial derivative with respect to the dummy force, the analytical results for the axial and vertical deflections are calculated. The outcome of the derived tapered beam model is compared to elementary results from Bernoulli-Euler and Timoshenko. The target solutions are obtained by two-dimensional finite element calculations for a tapered cantilever and a clamped-hinged beam subjected to various loads. It is shown that both the shear stress and also the transverse normal stress are correctly predicted by the new method and the deflections computed by Castigliano’s theorem are in a very good agreement with numerical solutions. Errors are significantly reduced compared to the errors of the Timoshenko solution.

## Introduction

Beam members with variable height are popular candidates for structural optimization algorithms when it comes to lightweight engineering topics like maximizing the strength, the stiffness and saving material. In contrast to prismatic beams, which have a straight center line and a non-varying cross section, beams with varying cross sections are a special topic in structural engineering where a better understanding based on improved analytical models is required. This contribution may be a starting point for deriving stiffness beam members for numerical calculations, to investigate orthotropic and anisotropic materials and layered tapered beam composites.

Non-prismatic and tapered beams are often considered as stepped beams with piecewise-constant cross sections in many textbooks in structural mechanics. Elementary beam theories (Bernoulli-Euler or Timoshenko) are applied to compute the deflections. But this approximation is misleading: it will be shown in this contribution that the shear and the transverse normal stress distributions between piecewise stepped beams with constant cross sections and tapered beams may significantly differ. Only for very thin beams, when variations of the cross section play a minor role, elementary theories may be applied. But significant differences to the Timoshenko theory occur for thicker structures with varying height because the well-known parabolic shear stress distribution, which is a second-order term for deflection curve, does not vanish over inclined surfaces, even if shear tractions do not occur. In other words, the zero-shear-stress condition is violated.

Unfortunately the nomenclature and the terminology in the scientific literature are ambiguous, when it comes to the classification of non-prismatic beams. On the one hand, one has to distinguish between constant cross sections orthogonal to the straight or curvilinear center lines. These examples are understood as *prismatic beams* and *curved beams*, respectively. A highly general beam with a curvilinear center line and a variable cross section is called *a non-prismatic beam*. A beam with variable cross section and a straight center-line is called *a beam of variable cross section*. However, this classification is often unclear or terms are simply mixed up. To the best knowledge of the author, a specific technical term for profiles with asymmetric cross sections (i.e., different tapering angles for lower and upper surfaces) and curvilinear center lines does not exist.

Classical structural mechanics books provide independent chapters for curved beams and those with variable cross sections, see Timoshenko and Goodier [1] and [2], Bruhns [3]. One of the first contributions that can be considered as a starting point addressing a new theory for beams of variable cross section, is the work by Boley [4]. Based on Boley’s earlier work on the accuracy of rectangular beams [5], he calculates the Airy stress function for nonuniform beams. Error estimations for the stresses and curvature are presented and solutions are compared to the wedge theory, see Timoshenko and Goodier [1] Art.38 and 45. Reissner [6] investigates shear lag of a box beam with tapered cover sheets. With the help of the least work theorem (Castigliano’s method) a second-order differential equation is obtained for the shear lag influence, leading to a better approximation of the axial stress in the flanges is obtained and the effective width is calculated. Several more box beams are studied to evaluate the influence of shear lag in Hildebrand and Reissner [7], i.e., various boundary conditions and even nonlinear height variations of the sheets are analyzed. The variational-asymptotic method is used to obtain an asymptotically-exact expression for the strain energy of a tapered beam, see Hodges et al. [8]: Depending on the tapering angle, asymptotically-exact section constants are then used to adapt section constants for a generalized Timoshenko beam theory. In a further study by Hodges et al. [9] the novel feature of the beam theory is that the effect of the taper parameter on the lateral-surface boundary conditions must be included. The accuracy of the cross-sectional stiffnesses was found to be in excellent agreement with elasticity solutions for extension, bending and flexure from Timoshenko and Goodier [1] and Krahula [10].

A dynamic analysis of a three-layer beam with interlayer slip, whose external layers are tapered, is performed by Adam et al. [11]. Natural frequencies, eigenfunctions and forced vibration responses are computed and compared to two-dimensional finite element results. In Beltempo et al. [12], the aim is to accurately predict displacements and stresses with a minimum number of unknowns. The dimensional reduction starts from the Hellinger-Reissner functional with displacements and stresses as independent variables. Finite elements are developed by Shooshtari and Khajavi [13] based on suitable modifications of Euler-Bernoulli or Timoshenko beam model coefficients. An element stiffness matrix based on the analytical beam solution of prismatic beams is derived by Mercuri et al. [14]. Additionally, comparisons with the structural and earthquake engineering software SAP2000 are performed, highlighting critical issues and severe errors in stress recovery of this widely used industrial software package. One possibility for stress recovery is Jourawski’s approach, see e.g., [15]. For sandwich structures this procedure has been thoroughly developed by Bardella and Mattei [16] and [17] to compute accurate stress distributions.

This paper aims to derive analytical formulae for the stress fields and the deflection curve of symmetrically tapered beams. Integrating the local form of the equilibrium equations and considering the correct stress boundary conditions over the inclined surfaces, accurate results for the stress distribution are obtained as functions of the internal beam forces and moments. The complementary strain energy is computed which serves as the basis of Castigliano’s second theorem. Adding dummy or phantom forces to the real load, the horizontal and vertical deflection curves as well as the rotation angle can be calculated. The analytical outcome is verified by finite element results for several beam configurations and loads. To demonstrate that elementary beam theories fail and that they yield inaccurate results, stress distributions and deflections are studied for these very simple beam configurations intentionally. The presented results show that for all benchmark problems under consideration, the relative error of the presented tapered beam theory (TBT) is significantly reduced compared to the Timoshenko-beam results. Results from two-dimensional finite element calculations are considered as target or reference solutions. Parameter studies confirm that for larger values of the height-to-length ratio and the tapering parameter, the error of the presented theory is in the range of one-tenth compared to elementary results. Because of the good correlation between analytical and numerical results, profiles with different upper and lower surfaces and asymmetric cross sections are investigated in an upcoming contribution.

**Fig. 1:**
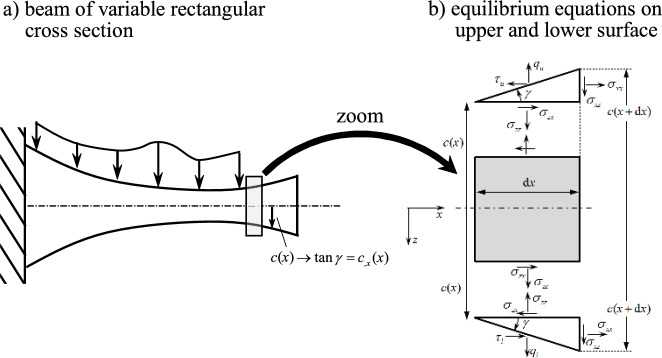
**a** Beam with variable rectangular cross section and **b** equilibrium triangles over the upper and the lower surface with normal ($q_l(x)$, $q_u(x)$) and shear components ($\tau _l(x)$, $\tau _u(x)$) of the surface traction

## Modeling of a beam with variable rectangular cross section

The equilibrium conditions for a two-dimensional problem read

1 $$\begin{aligned}  &   \sigma _{xx,x} + \sigma _{xz,z} = 0 \end{aligned}$$

2 $$\begin{aligned}  &   \sigma _{xz,x} + \sigma _{zz,z} = 0 \end{aligned}$$

Integrating both equations with respect to the beam height and weighting the first one by *z*, one obtains three equilibrium equations on beam level

3 $$\begin{aligned}  &   N_{,x} + \tau _{l} - \tau _{u} = 0 \end{aligned}$$

4 $$\begin{aligned}  &   Q_{,x} + q_{l} - q_{u} = 0 \end{aligned}$$

5 $$\begin{aligned}  &   M_{,x} - Q + \left( \tau _{l} + \tau _{u} \right) c = 0 \end{aligned}$$

where $$[N,\,Q,\,M]^T = \int _{-c}^{c} [ \sigma _{xx},\, \sigma _{xz},\, \sigma _{xx} z ]^T b \, \textrm{d}z$$ are the axial force *N*(*x*), the shear force *Q*(*x*) and the bending moment *M*(*x*). The width is denoted by *b*, the varying half height by *c*(*x*) from which the cross-sectional area *A* and the moment of inertia *I* follow as $$A=2 b c$$ and $$ I = 2 b c^3/3$$. The shear stress and the transverse normal stress over the lower and upper surfaces (multiplied by the width *b*) are $$\tau _l$$, $$\tau _u$$, $$q_l$$ and $$q_u$$, respectively. For the sake of completeness, the axial force, the shear force and the bending moment read

6 $$\begin{aligned}  &   N(x) = \int _{-c(x)}^{c(x)} \sigma _{xx} b \, \textrm{d}z \end{aligned}$$

7 $$\begin{aligned}  &   Q(x) = \int _{-c(x)}^{c(x)} \sigma _{xz} b \, \textrm{d}z \end{aligned}$$

8 $$\begin{aligned}  &   M(x) = \int _{-c(x)}^{c(x)} \sigma _{xx}z b \, \textrm{d}z \end{aligned}$$

### Equilibrium of stresses and loads over the upper and lower surface

Figure 1 shows a symmetric beam with the total variable height 2*c*(*x*). The distributed loads and shear tractions over the lower and upper surface are denoted as $q_l (x)$, $q_u (x)$, $\tau _l (x)$ and $\tau _u (x)$, respectively. It can be shown by analytical considerations for prismatic beams (e.g., Boley and Tolin [5]) that the order of magnitude of the axial stress $\sigma _{xx}$ is the more dominant stress term compared to the shear stress $\sigma _{xz}$ and the transverse normal stress $\sigma _{zz}$. Integrating Eq. (1) with respect to *x*, the shear stress is computed if the boundary conditions are known at $z=\pm c(x)$. Considering the upper triangle at $z = -c(x)$ in Fig. 1b, it can be shown that the shear stresses $\sigma _{xz}$ and $\sigma _{zx}$ are equal in the triangle: the balance of linear momentum for the upper triangle yields

9 $$\begin{aligned} \sigma _{zx} \textrm{d}x \frac{ \textrm{d}z }{ 2 } - \sigma _{xz} \textrm{d}z \frac{ \textrm{d}x }{ 2 } = 0 \quad \textrm{with} \quad \textrm{d}z =\textrm{d}x \tan \gamma \qquad \rightarrow \qquad \sigma _{xz} = \sigma _{zx} \end{aligned}$$

It is noted that this equality does not hold if a distributed moment load per unit length (often referred to as $m_z$) acts over the surface. The equilibrium relations in the *x* and the *z*-direction read

10 $$\begin{aligned}  &   \sigma _{xx} \tan \gamma + \sigma _{xz} - \frac{\tau _u}{b} = 0 \end{aligned}$$

11 $$\begin{aligned}  &   \sigma _{xz} \tan \gamma + \sigma _{zz} - \frac{q_u}{b} = 0 \end{aligned}$$

Geometrical relations show that the local tapering angle $\gamma (x)$ is related to the inclined surface by $\tan \gamma = c_{,x}$. If the height decreases linearly

12 $$\begin{aligned} c(x) = c_0 \left( 1-\alpha \frac{x}{l} \right) \qquad \rightarrow \qquad \tan \gamma = -\alpha \frac{c_0}{l} \end{aligned}$$

the tapering angle $\gamma$ is constant and negative. For the derivation of more simplified relations the normalized axial force $N^*(x)$ with respect to the cross-sectional area *A*(*x*) and the normalized bending moment $M^*(x)$ with respect to the geometric moment of inertia *I*(*x*) are introduced

13 $$\begin{aligned} N^*(x) = \frac{ N(x) }{ A(x) } \qquad \qquad M^*(x) = \frac{ M(x) }{ I(x) } \end{aligned}$$

One fundamental assumption in this contribution is that the axial stress $\sigma _{xx}$ depends on a constant part due to the normal force *N*(*x*) and a linearly varying part due to the bending moment *M*(*x*). From elementary beam theory it holds for prismatic beams

14 $$\begin{aligned} \sigma _{xx} = N^*(x) + M^*(x) z \end{aligned}$$

Substituting this elementary relation for the axial stress (14) into Eqs. (10) and (11) and solving for the shear and the transverse normal stress at $z=-c$, one finds

15 $$\begin{aligned}  &   \sigma _{xz}(x,-c) = - \left[ N^*(x) - M^*(x) c \right] \tan \gamma + \frac{\tau _u}{b} \end{aligned}$$

16 $$\begin{aligned}  &   \sigma _{zz}(x,-c) = \left[ N^*(x) - M^*(x) c \right] \tan ^2 \gamma - \frac{\tau _u}{b} \tan \gamma + \frac{q_u}{b} \end{aligned}$$

One observes from Eq. (15) that the shear stress does not only depend on the shear traction $\tau _u$ at the upper surface, but also on the local axial stress and the tapering angle $\gamma$. This means that the *zero-shear-stress condition* for traction-free prismatic beams does not hold for tapered beams. Consequently, Timoshenko-like beam theories for varying cross sections have to consider an *x*-axis dependent shear correction factor, because the shear stress profile is varying in both the *x* an *z*-axis and will not have the well-known parabolic shape that fulfills the zero-shear-stress condition, see Prescott [18], Cowper [19], Stephen and Levinson [20] and Schoeftner [21].

The equilibrium relations over the lower surface at $z = c(x)$ read

17 $$\begin{aligned}  &   \sigma _{xz} - \sigma _{xx} \tan \gamma - \frac{\tau _l}{b} = 0 \end{aligned}$$

18 $$\begin{aligned}  &   \sigma _{zz} - \sigma _{xz} \tan \gamma - \frac{q_l}{b} = 0 \end{aligned}$$

Inserting the axial stress Eq. (14) into these two relations again, one finds

19 $$\begin{aligned}  &   \sigma _{xz}(x,c) = \left[ N^*(x) + M^*(x) c \right] \tan \gamma + \frac{\tau _l}{b} \nonumber \\  &   \sigma _{zz}(x,c) = \left[ N^*(x) + M^*(x) c \right] \tan ^2 \gamma + \frac{\tau _l}{b} \tan \gamma + \frac{q_l}{b} \end{aligned}$$

## Calculation of the shear stress and the transverse normal stress

### Analytical relation between the shear stress, the height and the internal beam forces

The shear stress is calculated by Jourawski’s approach, see e.g., [15] or in the context of stress estimation by post-processing, the reader is referred to Bardella and Mattei [16] and [17]. Substituting Eq. (14) into Eq. (1) and integrating with respect to *z*, one finds for the shear stress

20 $$\begin{aligned} \sigma _{xz} = - M^*_{,x}(x) \frac{z^2}{ 2 } - N^*_{,x}(x) z + C_0 (x) \end{aligned}$$

The following three possibilities exist to calculate the *x*-dependent function $C_0 (x)$:

- the shear stress equals Eq. (19) at the upper layer $z = -c(x)$, see Fig. 1b.
- the shear stress equals Eq. (19) at the lower layer $z = c(x)$, see Fig. 1b.
- integrating Eq. (20) with respect to the cross-sectional area equals the shear force *Q*(*x*).

Of course, it can be shown that these three possibilities are equivalent. The third possibility is preferred because it allows for an easier interpretation of the results. Substituting Eq. (20) into the shear force relation (7), one finds the unknown function $C_0 (x)$

21 $$\begin{aligned} C_0 (x) = Q^*(x) + M^*_{,x}(x) \frac{ c^2(x) }{ 6 } \qquad \textrm{with} \qquad Q^*(x) = \frac{ Q(x) }{ A(x) } \end{aligned}$$

where the normalized shear force $Q^*(x)$ equals the mean shear stress at location *x*. The final solution of the shear stress is obtained by inserting Eq. (21) into Eq. (20)

22 $$\begin{aligned} \sigma _{xz} = Q^*(x) - N^*_{,x}(x) z - M^*_{,x}(x) \left( \frac{z^2}{ 2 } - \frac{c^2(x)}{ 6 } \right) \end{aligned}$$

Equation (22) shows that the shear stress equals the mean shear stress $Q^*(x)$ plus terms which are related to the bending moment and the normal force. The latter terms are self-equilibrated: they consider stress variations in the height direction but their resultants are zero. In case of a prismatic beams with $c'(x) = 0$, vanishing shear tractions $\tau _l = \tau _u = 0$ and $M_{,x} = Q$ (see Eq. (5)), one obtains the parabolic shear stress distribution $\sigma _{xz} = \frac{3}{2} Q^*(x) \left( 1 - \frac{z^2}{ c^2 } \right)$.

### Analytical relation between the transverse normal stress, the height and the internal beam forces

Taking into account Eq. (22) one finds for the transverse normal stress by integrating Eq. (2) with respect to *z*

23 $$\begin{aligned}  &   \sigma _{zz} = - \int _{ -c(x) }^{ c(x) } \sigma _{xz,x} \, \textrm{d}z + D_0(x) = N^*_{,xx}(x) \frac{z^2}{2} + M^*_{,xx}(x) \left( \frac{z^3}{ 6 } - \frac{c^2(x) z}{ 6 } \right) \nonumber \\  &   \qquad \quad - M^*_{,x}(x) \frac{ c(x) c_{,x}(x) z }{3} - Q^*_{,x}(x) z + D_0(x) \end{aligned}$$

Considering the boundary conditions (16) or (19), the function $D_0(x)$ is computed and the solution for the transverse normal stress $\sigma _{zz}$ reads

24 $$\begin{aligned}  &   \sigma _{zz} = N^*_{,xx}(x) \left[ \frac{z^2}{2} - \frac{c^2(x)}{2} \right] - N^*_{,x}(x) c(x) c_{,x}(x) + M^*_{,xx}(x) \left[ \frac{z^3}{ 6 } - \frac{c^2(x) z}{ 6 } \right] \nonumber \\  &   \qquad \quad - M^*_{,x}(x) \frac{ c(x) c_{,x}(x) z }{3} + Q^*_{,x}(x) \left[ c(x) - z \right] + Q^*(x) c_{,x}(x) + \frac{q_l(x)}{b} \end{aligned}$$

Of course, this result includes the transverse normal stress distribution for prismatic beams. Assuming a beam loaded by $q_l(x)$ and $q_u(x)$ (i.e., $\tau _u(x) = \tau _l(x) = 0$), when $M_{,xx} = -q_l + q_u$ holds, one finds for the prismatic beam

25 $$\begin{aligned} \sigma _{zz} = M^*_{,xx}(x) \left( \frac{z^3}{ 6 } - \frac{c^2 z}{ 2 } + \frac{c^3}{3} \right) + \frac{q_l(x)}{b} \end{aligned}$$

One can easily check that the boundary conditions $\sigma _{zz}(x,-c) = q_u(x)$ and $\sigma _{zz}(x,c) = q_l(x)$ are fulfilled by Eq. (25), see also Boley-Tolins [5] and Schoeftner [24].

It is noted that even more accurate, higher order stress distributions could be found by inserting Eqs. (14), (22) and (24) into the compatibility equation to calculate a higher order term for the axial stress (=deviations from the linear or constant parts in Eq. (14)). This procedure would be similar to the Boley-Tolins iteration method.

## Calculation of the horizontal and vertical deflection by Castigliano’s theorem

Castigliano’s method is a suitable approach to calculate the deflection, the rotation angle and the axial deflection if the stress components are known as a function of the applied load and so-called dummy or phantom forces and moments. The main advantage of these dummy loads is that the *x*-dependent deflection curves can be computed, because their values approach zero in the final step, see Dym [22], Bao [23] or some recently published works by the author on higher order beam theories [24] and [25]. Although Castigliano’s method is hardly used nowadays in recent scientific publications and considered to be old-fashioned and out-of-date, we will see in Sect. 5 that highly accurate analytical results are obtained for all benchmark examples. Additionally, a main advantage is that shear correction factors are not required because the exact shear stress $\sigma _{xz}$ (and even the transverse normal stress $\sigma _{zz}$) is known, see Eqs. (22) and (24).

### Short step-by-step explanation of the analytical results

**Fig. 2:**
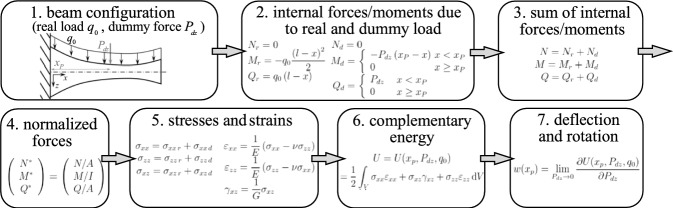
Derivation of the analytical results in Appendix A via Castigliano’s theorem in seven steps

A detailed derivation of the results is not given in this contribution due to lengthy cumbersome mathematical expressions. By explaining the necessary steps (Fig. 2), the interested reader is able to verify the analytical outcome by using the symbolic software tool MATHEMATICA or MAPLE. Analytical results for the deflection curve and the axial deflection are given by Eqs. (A1)–(A23).

- STEP 1: the actual tapered beam configuration is shown in Fig. 2, e.g., a symmetric configuration is considered subjected to the distributed real load $q_0$. The dummy force $P_{dz}$ is located at $x=x_p$.
- STEP 2: the axial force *N*(*x*), the shear force *Q*(*x*) and the bending moment distribution *M*(*x*) as functions of the real load (subscript *r*) and the dummy forces (subscript *d*) are computed.
- STEP 3: the real and dummy forces and moments are added.
- STEP 4: the normalized internal beam forces $N^*(x)$, $Q^*(x)$, $M^*(x)$ are computed, see Eqs. (13) and (21), which depend on the real load and the dummy forces.
- STEP 5: these results are inserted into the expressions for the axial stress $\sigma _{xx}$, the shear stress $\sigma _{xz}$ and the transverse normal stress $\sigma _{zz}$, see Eqs. (14), (22) and (24).
- STEP 6: the stresses are inserted into the complementary energy *U*.
- STEP 7: the deflection are calculated (either the axial deflection $u_0$, the vertical deflection *w* or the rotation angle $\varphi$) by computing the partial derivative of the complementary energy with respect to the dummy force or moment.

It is noted that the height *c*(*x*) can be arbitrarily chosen, i.e., the results derived from STEP 1–7 hold for beams with constant cross section, for tapered beams and for beams as those depicted in Fig. 1 (e.g., profile with parabolic height distribution). In the following analytical results for a tapered beam with (half-) height $c(x) = c_0 (1-\alpha x/l)$ are given. The beam is subjected to the end moment $M_0$, the tip force $F_0$ and the constant distributed load $q_0$ in Sect. (A.1), Sect. (A.2) and Sect. (A.3), respectively. For a more detailed description how to derive higher order beam formulae via Castigliano’s theorem, the reader is referred to Schoeftner and Gahleitner [25], where prismatic orthotropic beams are investigated.

## Numerical benchmark examples and verification with finite elements

As benchmark examples a cantilever and a clamped-hinged beam are studied. Three different load cases are assumed for the tapered cantilever (either an end moment (Sect. 5.1), a tip force (Sect. 5.2) or a constant distributed load (Sect. 5.3)), whereas for the clamped-hinged beam a distributed load is considered (Sect. 5.4). For all these configurations the tapering parameter is $\alpha =0.4$, i.e., the total height is $c(0) = 0.16 \, \textrm{m}$ at $x=0$ and $c(l) = 0.096 \, \textrm{m}$ at $x=l$, see Eq. (12) and Table 1. The height-to-length ratio at $x=0$ (clamped end) is $\lambda = 2c_0/l = 1/5$ and the beam width is constant $b = 0.01 \textrm{m}$.

Finally, variations of the height-to-length ratio $\lambda$ ranging from very thick beams ($\lambda =1/2$) to thin beams ($\lambda =1/10$) are presented in Sect. 5.5: the tapering parameter is either $\alpha = 4/10$ (gentle taper) or $\alpha = 99/100$ (sharp taper). The geometrical and material parameters are summarized in Table 1. Results from the tapered beam theory (Sect. 4) are compared to target solutions from finite element calculations and analytical results obtained by the Bernoulli-Euler and the Timoshenko theory. It is noted that a shear correction factor $\kappa =5/6$ is assumed for the Timoshenko theory. Although this value is only an approximation (see Cowper [19], Boley [5] or Schoeftner [21]), it is a commonly excepted result that considers the parabolic shear stress distribution for prismatic beams. However, for tapered beams the zero-shear-stress condition at $z=\pm c(x)$ is violated and the shear correction factor may be modified. Two-dimensional finite element (FE) results are considered as target solutions: A Q8-element (eight-node quadrilateral element with quadratic shape functions) for the displacement nodes is used for the finite element computations. The FE-formulation is programmed in MATLAB and the author uses a modified code based on Ferreira’s book [26]. For the numerical examples (Sect. 5.1–5.4), 72 elements in the *x*-direction and 28 elements in the *z*-direction are used, yielding 8265 nodes for the Q8-mesh. Hence, the total number of degrees of freedom for the two-dimensional plane-stress problem is 16530.

**Table 1:** Parameters for the numerical examples

Variable (unit)	Value	Description
$E \,\quad (\textrm{Nm}^{-2})$	$2.1 \cdot 10^{11}$	Young’s modulus
$\nu \,\quad (\mathrm {-})$	0.3	Poisson ratio
$l \,\quad (\textrm{m})$	0.8	length
$c_0 \,\quad (\textrm{m})$	0.08	half of the height at $x=0$
$\alpha \,\quad (\mathrm {-})$	tapering parameter	
$c(x)=c_0(1-\alpha x/l) \,\quad (\textrm{m})$	Varying	half of the height depends on $\alpha$
$\lambda = c_0/l \,\quad (\mathrm {-})$	0.2	height-to-length ratio at $x=0$
${ b \,\quad (\textrm{m}) }$	0.01	beam width

### Tapered cantilever with linearly decreasing height and end moment

The first example is a cantilever subjected to the end moment $M_0 = 1000~\textrm{Nm}$ (in the positive *y*-direction, hence the deflection will be negative according to the coordinate system in Fig. 1b). The tapering parameter $\alpha = 4/10$.

**Fig. 3:**
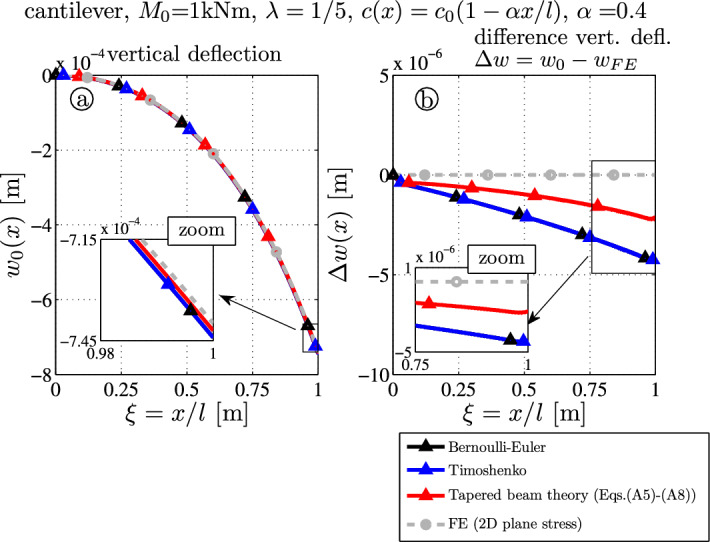
Tapered thick cantilever ($\lambda = 1/5$) with end moment $M_0$: **a** deflection curves $w_0$; **b** error of the deflection curve from the FE target solution $\Delta w(x) = w_0(x) - w_{FE}(x,z=0)$

Figure 3a shows the deflection curve. The colored results of the analytical theories are as follows:

- Bernoulli-Euler (BE-black),
- Timoshenko (TS-blue),
- tapered beam theory with Castigliano’s theorem (TBT-red), see Eq. (A1).

These results are compared to the finite element outcome (FE-gray). The difference to the finite element result is denoted as error (Fig. 3b)

26 $$\begin{aligned} e_i(x) = w_i (x) - w_{FE}(x,z=0) \qquad \textrm{with} \qquad i = \mathrm {BE,\,\, TS\,\, or \,\, TBT} \end{aligned}$$

One observes that the deflection curve of all four curves are approximately the same and differences are hardly recognizable (Fig. 3a): the qualitative curve is a parabola, but the error definition and Fig. 3b give a better insight into the quantitative results of the various theories.

The tip deflections read:

- $7.440 \times 10^{-4} \, \textrm{m}$ (BE-black and TS-blue),
- $7.420 \times 10^{-4} \, \textrm{m}$ (TBT-red) and
- $$7.398 \times 10^{-4} \, \textrm{m}$$ (FE-gray).

It is noted that for the deflection of the FE-results $w_{FE}(x,z=0)$ is used for comparison, and not the cross section averaged value $w_{FE \, avg.}(x) = \int _{-c}^{c} w_{FE}(x,z) \, \textrm{d}z$. These values slightly differ and read $w_{FE}(l,0) = 7.398 \times 10^{-4} \, \textrm{m}$ and $w_{FE \, avg.}(l) = 7.409 \times 10^{-4} \, \textrm{m}$ at $x=l$.

From Fig. 3b it becomes clear that even for this simple example the error is reduced by $50 \, \%$ by tapered beam theory (TBT): for the Bernoulli and the Timshenko theory the error is $e_{BE}(x) = e_{TS}(x) = -4.2 \times 10^{-6} \, \textrm{m}$, but for TBT it is only $e_{BE,\, TS} = -2.2 \times 10^{-6} \, \textrm{m}$.

**Fig. 4:**
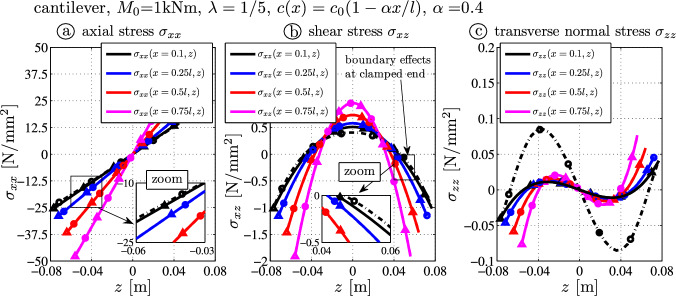
Stress distributions due to the end moment $M_0 (x)$ – comparison of FE target solutions ($-\bigcirc -$) and the analytical TBT-results ($-\triangle -$): **a** axial stress $\sigma _{xx}$ (14), **b** shear stress $\sigma _{xz}$ (22), **c** transverse normal stress $\sigma _{zz}$ (24)

Figure  4a, b and c shows the axial, the shear and the transverse normal stress distributions, respectively. The selected locations are $x=0.1 l$ (close to the clamped end – black), $x=0.25 l$ (blue), $x=0.50 l$ (red) and $x=0.75 l$ (cyan). While the axial stress is the trivial linear solution, one observes the nonzero shear stress over the surfaces at $z = \pm c(x)$ (Fig. 4b), although no loads act on these surfaces. For this load case $N(x)=0 = N^*(x) = 0 = Q(x) = Q^*(x)=0$ holds: Despite $M(x) = M_1 = \mathrm {const.}$, the variation of the normalized moment does not vanish ($M^*_{,x}(x) \ne M_{,x}(x) = 0$ because of $I_{,x}(x) \ne \mathrm {const.}$) and consequently the last term in Eq. (22) does not vanish locally. The analytical (TBT) and numerical (FE) results are in very good agreement in general. Only close to the clamped-end a non-trivial state of stress occurs due to the boundary constraints in the finite element model (black curve, $x=0.1l$).

The transverse normal stress $\sigma _{xz}$ is shown in Fig. 4c. Differences are only visible close to the kinematic constraints (black curve, $x=0.1l$), but at the other selected locations at $x=\{0.25l, \, 0.5l,\, 0.75 l \}$, these lower order stress components match with the numerical results. Summing up, when zooming into the figures (as it is shown in Fig. 4a, b) the stress results from the analytical relations match very well with the FE-results.

### Tapered cantilever with linearly decreasing height and tip force

**Fig. 5:**
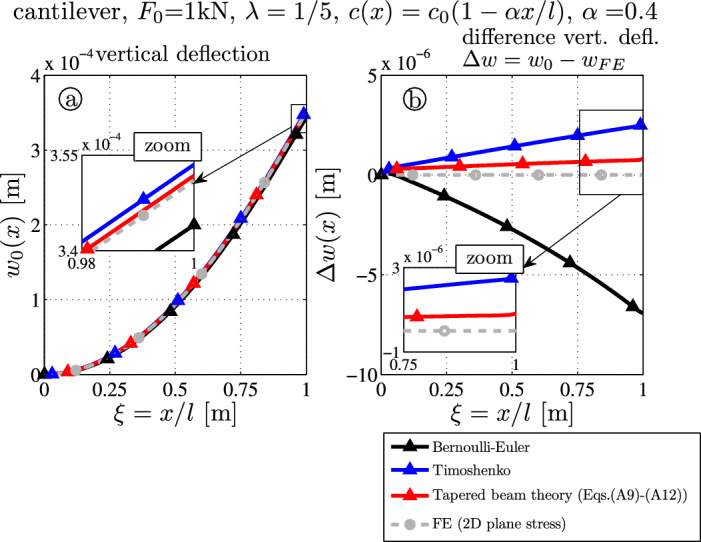
Tapered thick cantilever ($\lambda = 1/5$) with tip force $F_0$: **a** deflection curves $w_0$; **b** error of the deflection curve from the FE target solution $\Delta w(x) = w_0(x) - w_{FE}(x,z=0)$

For the second example, the tip force $F_0 = 1000~\textrm{N}$ acts on the tapered beam. Figure 5a and b shows the deflection curve and the deflection error.

The tip deflections read:

- $$3.440 \times 10^{-4} \, \textrm{m}$$ (BE-black),
- $$3.535 \times 10^{-4} \, \textrm{m}$$ (TS-blue),
- $3.517 \times 10^{-4} \, \textrm{m}$ (TBT-red) and
- $$3.510 \times 10^{-4} \, \textrm{m}$$ (FE-gray).

It follows that the tip deflection error of the presented theory (TBT) is only $0.20\%$, but for the elementary results it is $0.71\%$ (TS) and $-2.00\%$ (BE). This shows the potential of the presented theory where the residual error is less than one third of the error from the Timoshenko model.

**Fig. 6:**
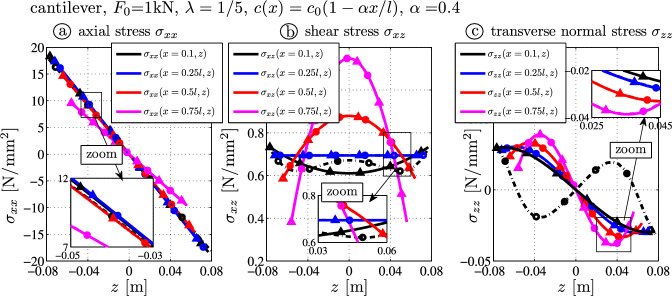
Stress distributions due to the tip force $F_0 (x)$ – comparison of FE target solutions ($-\bigcirc -$) and the analytical TBT-results ($-\triangle -$): **a** axial stress $\sigma _{xx}$ (14), **b** shear stress $\sigma _{xz}$ (22), **c** transverse normal stress $\sigma _{zz}$ (24)

Figure 6a, b and c shows the stress distributions at $x=0.1 l$ (black, close to the clamped end), at $x=0.25 l$ (blue), at $x=0.50 l$ (red) and at $x=0.75 l$ (cyan). Again, one observes the nonzero shear stress over the upper and lower surfaces (Fig. 6b) by substituting $N^*(x)=0$, $Q^*(x)=-F_0/A(x)$ and $M^*(x) = -F_0 (x-l)/I(x)$ into Eq. (22). The stress distributions are in good agreement between FE and analytical TBT-results, but minor differences are caused by the kinematic constraints and visible in the surrounding only (black curve at $x=0.1l$). One notes that the transverse normal stress $\sigma _{zz}$ is also nonzero although distributed loads are not present at $z=\pm c(x)$ (Fig. 6c and Eq. (24)): FE and TBT results are in good agreement except for the stresses close to $x \approx 0$.

### Tapered cantilever with linearly decreasing height and distributed load

The third benchmark example represents a tapered beam with constant distributed load $q_0 = 1000\mathrm {N/m}$ over the lower surface (i.e., $z=c$). Figure 5a and b shows the deflection curve and the deflection error.

**Fig. 7:**
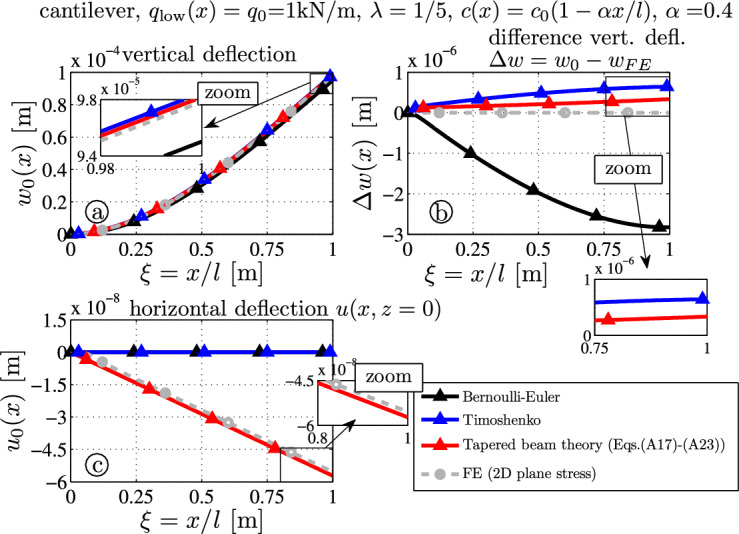
Tapered thick cantilever ($\lambda = 1/5$) with constant load $q_l (x) = q_0$: **a** deflection curves $w_0$; **b** error of the deflection curve from the FE target solution $\Delta w(x) = w_0(x) - w_{FE}(x,z=0)$; **c** axial deflection $u_0$ at $z=0$

The tip deflections (Fig. 7a) read

- $$9.502 \times 10^{-5} \, \textrm{m}$$ (BE-black),
- $$9.850 \times 10^{-5} \, \textrm{m}$$ (TS-blue),
- $9.818 \times 10^{-5} \, \textrm{m}$ (TBT-red) and
- $$9.785 \times 10^{-5} \, \textrm{m}$$ (FE-gray).

The tip deflection error of the presented theory (TBT) is $0.34\%$, which is half of the error of the Timoshenko theory $0.66\%$ (compare the red and blue curves in Fig. 7b). The Bernoulli-Euler theory underestimates the results and the tip deflection error is $2.9\%$. Additionally, the axial deflection $u_0(x)$ at $z=0$ is shown in (Fig. 7c). According to the analytical TBT-theory (red), which is the only theory with a nonzero result, it is a linearly decreasing line with $u_0(l) = -5.72 \times 10^{-8} \, \textrm{m}$ and very close to the FE results $u_{FE}(l,0) = -5.54 \times 10^{-8} \, \textrm{m}$, see Eqs. (A21) and (A28). It is noted that most beam theories do not yield this small deflection which shows that the neutral fiber does not coincide with the symmetry axis at $z=0$, see the results for rectangular isotropic or orthotropic beams in Schoeftner [21, 24, 25]. This non-vanishing axial deflection is caused by the transverse normal stress, the Poisson ratio $\nu$, the height-to-length ratio $\lambda$ and the tapering parameter $\alpha$: from Eq. (A21) one observes that the neutral fiber is located at $z=0$ only in case of $\alpha = \sqrt{12 \nu /\lambda }$ (without showing here, this can be verified by the FE-results).

**Fig. 8:**
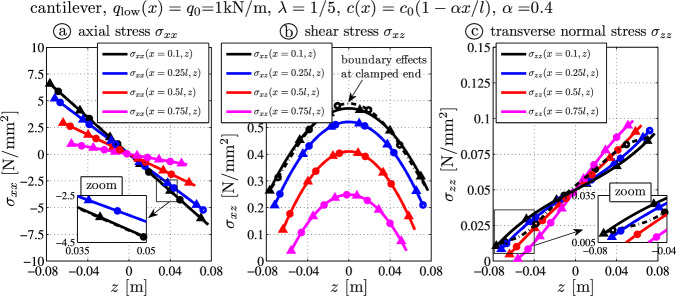
Stress distributions due to the constant load $q_l (x) = q_0$ – comparison of FE target solutions ($-\bigcirc -$) and the analytical TBT-results ($-\triangle -$): **a** axial stress $\sigma _{xx}$ (14), **b** shear stress $\sigma _{xz}$ (22), **c** transverse normal stress $\sigma _{zz}$ (24)

Figure  8 shows the stress distributions $\sigma _{xx}$, $\sigma _{xz}$ and $\sigma _{zz}$. Results from the presented tapered beam theory (TBT) are in good correlation with the numerical outcome. One observes the nonzero boundary shear stress at $z=\pm c(x)$ (Fig. 8b) and the coinciding curves for the transverse normal stress $\sigma _{zz}$ (Fig. 8c): neglecting this lower order stress component in the complementary energy (Fig. 2) would mean a vanishing axial deflection at $z=0$.

### Clamped-hinged beam with distributed load and linearly decreasing height

The fourth benchmark example is a clamped-hinged tapered beam. Its deflection is caused by a constant distributed load $q_0 = 1000 \mathrm {N/m}$ over the lower surface (i.e., $z=c$). Results for this statically indeterminate configuration cannot be directly solved by Castigliano’s theory, but results from Sects. 5.3 and 5.2 are superposed: first the kinematic constraint at $x=l$ (=the hinge) is released and the beam is loaded by the yet unknown redundant force $F_0$. Then the value of $F_0$ is computed in such a manner that the kinematic constraint $w(l) = 0$ is fulfilled.

**Fig. 9:**
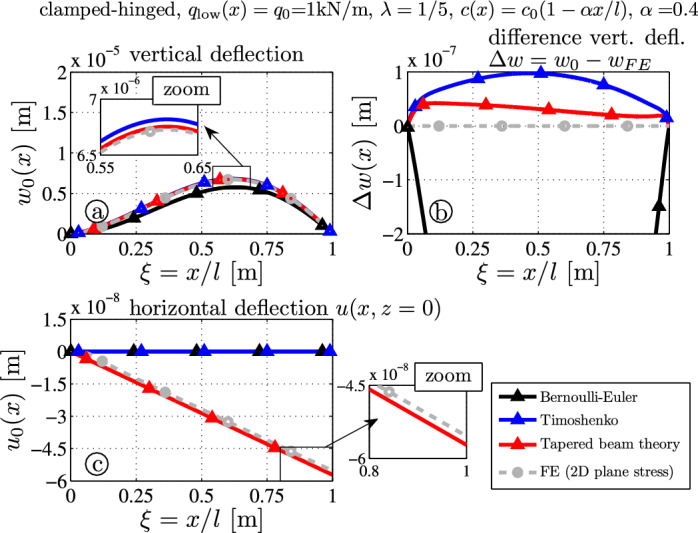
Tapered thick clamped-hinged beam ($\lambda = 1/5$) with constant load $q_l (x) = q_0$: **a** deflection curves $w_0$; **b** error of the deflection curve from the FE target solution $\Delta w(x) = w_0(x) - w_{FE}(x,z=0)$; **c** axial deflection $u_0$ at $z=0$

One observes that the maximal deflection at $\xi \approx 0.62$ (Fig. 9a) is underestimated by the BE beam (black, $w_{0\text{ max }} \approx 5.77 \times 10^{-6} \, \text {m}$) and overestimated by the Timoshenko theory (blue, $w_{0 \text{ max }} \approx 6.82 \times 10^{-6} \, \text {m}$). The largest deflection is approximately the same for the analytical theory (TBT-red, $w_{0 \text{ max }} \approx 6.75 \times 10^{-6} \, \text {m}$) and the FE outcome (gray, $w_{\text{ FE } \, \text{ max }} \approx 6.73 \times 10^{-6} \, \text {m}$). Hence the average deflection error is reduced by $75\%$ compared to the Timoshenko theory (cf. difference between blue and red line in Fig. 9b). Even the axial deflections of TBT and FE-results are very close: $u_{0}(l) \approx -5.72 \times 10^{-8} \, \textrm{m}$ and $u_{\text { FE}}(l,0) \approx -5.54 \times 10^{-8} \, \textrm{m}$.

**Fig. 10:**
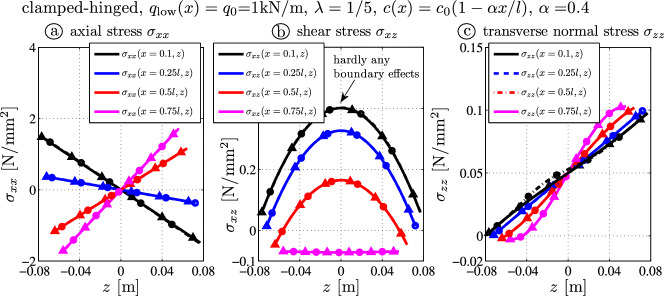
Stress distributions due to the constant load $q_l (x) = q_0$ for a clamped-hinged tapered beam – comparison of FE target solutions ($-\bigcirc -$) and the analytical TBT-results ($-\triangle -$): **a** axial stress $\sigma _{xx}$, **b** shear stress $\sigma _{xz}$, **c** transverse normal stress $\sigma _{zz}$

Fig. 10 shows the stress distributions. It is interesting to see that at $x=0.75l$ the shear stress is constant. According to Eq. (22) this occurs when the following condition

27 $$\begin{aligned} M^*_{,x}(x) = 0 \rightarrow \frac{ M_{,x}(x) }{ I_{,x}(x) } = \frac{ M(x) }{ I(x) } \end{aligned}$$

holds. Then the *z*-dependent term in Eq. (22) is zero and $\sigma _{xz} = Q^* (x)$ does not depend on the *z*-axis, i.e., the shear stress equals the normalized shear force (=mean shear stress).

**Fig. 11:**
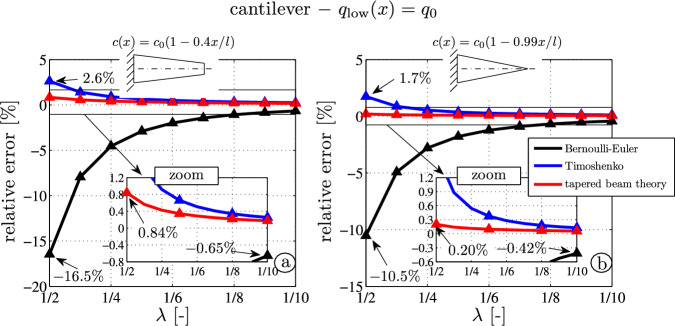
Relative error of the vertical deflection as a function of the height-to-length ratio $\lambda$: **a** moderately tapered cantilever $c(x) = c_0 (1-0.4 x/l)$ and **b** sharp-angled cantilever $c(x) = c_0 (1-0.99 x/l)$

### Parameter variations – Cantilever beam with constant distributed load

Finally, parameter variations show the accuracy of the derived tapered beam theory (TBT). A moderately tapered cantilever (Fig. 11a) and a sharp-tapered cantilever (Fig. 11b) are investigated. Both beams are subjected to a constant distributed load $q_0$. The beam height-to-length ratio varies from very thick ($\lambda = 1/2$) to thin beams ($\lambda = 1/10$). The relative tip deflection errors of BE (black), TS (blue) and TBT (red) are shown.

One observes that the error of TBT is only $0.2 \%$ for the sharp-tapered beam (Fig. 11b), even if the initial height-to-length ratio is very large (i.e., $\lambda = 1/2$). Although the Timoshenko beam yields an error of only $1.7\%$ (see the zoom window in Fig. 11b), which might be acceptable for most practical application, it should be kept in mind that the main disadvantage and deficiency of the Timoshenko theory for tapered beams are the misleading results for the shear stress. Furthermore, the transverse normal stress cannot be calculated at all. Just for comparison, the error of the BE-model is $10.5 \%$. For very thin beams $1/\lambda > 10$, the Bernoulli theory yields satisfying results (the deflection is underestimated, the error is $-0.42 \%$). The main conclusions from the parameter variations are that the analytical results obtained by Castigliano’s theory are in a much better agreement with the numerical FE-outcome than the Timoshenko (blue) and Bernolli-Euler (black) results (i.e., the red lines in Fig. 11a and b are very close to zero).

It is also interesting to see that the outcome of the analytical models are less accurate for larger tapering angles than the outcome for smaller tapering angles. Figure 11a and b show that for $\alpha = 0.99$ the Timoshenko theory yields an error of about $1.7 \%$. Contrary for $\alpha = 0.4$ the error is larger and reads $2.6 \%$. Similar conclusions hold for BE. One observes for TBT that the error is $0.84 \%$ only, but four times higher than for $\alpha = 0.99$. The author notices that replacing the cantilever beam by a clamped-hinged beam, or generally speaking by a statically indeterminate beam, these errors become larger. For sake of brevity, these results are not shown in this contribution.

## Conclusion

This study investigates stress distributions and deflections in tapered beams. By applying Jourawski’s theory, shear and transverse normal stresses are calculated based on assumptions about the axial stress. It is found that even without surface tractions, the vanishing shear stress conditions are violated and the boundary stress depends on local variations in height. Using Castigliano’s second theorem and fictitious forces, analytical expressions for the horizontal and vertical deflections are derived. Results are compared to the Bernoulli-Euler and Timoshenko theories. Finite element computations of a tapered cantilever and a clamped-hinged beam under various loads confirm that the proposed method accurately predicts shear and transverse normal stresses. Additionally, the computed deflections closely align with numerical solutions, showing significantly reduced errors in comparison with the Bernoulli-Euler and Timoshenko solution.

## Appendix A Analytical deflection curve

In the section analytical results for the deflection curve *w*(*x*) are presented for a tapered cantilever subjected to an end moment (Sect. A.1), a tip force (Sect. A.2) and a constant distributed load (Sect. A.3). The non-dimensional coordinate is introduced $\xi = x/l$. The axial deflection $u_0(x)$ is nonzero in case of $q_0 \ne 0$ only, when the nonzero transverse normal stress $\sigma _{zz}$ causes a shrinking or compression of the *x*-axis. The calculation procedure is shown in Fig. 2 (Sect. 4). For a better interpretation of the results the deflection *w*(*x*) is split up into three terms: the fundamental term is denoted as $w_{BE}(x)$ and caused by the square of the axial stress $\sigma _{xx}^2$. The shear dependent term $w_{shear}(x)$ is caused by $\sigma _{xz}^2$ and further higher order terms due to the product $\sigma _{xx} \sigma _{zz}$ are denoted by $w_{HO}(x)$.

Due to the lengthy expressions it is advised to use a symbolic mathematical software package (like MATHEMATICA or MAPLE) in order to execute these computations automatically.

### A.1 Analytical deflection curve of a tapered cantilever with end moment

Taking into account the bending moment and the shear force distributions $M(x) = M_0$ and $Q(x) = 0$ and following the procedure in Sect. 4 and Fig. 2 by introducing dummy force method, Castigliano’s theorem yields for the vertical deflection

A1 $$\begin{aligned}  &   w (x) = w_{BE}(x) + w_{\textrm{shear}}(x) + w_{\textrm{HO}}(x) \end{aligned}$$

A2 $$\begin{aligned}  &   w_{BE}(x) = -\frac{ M_0 l^2 }{ 2 E I(x) } \xi ^2 \left( 1-\alpha \xi \right) ^2 \end{aligned}$$

A3 $$\begin{aligned}  &   w_{shear}(x) = \frac{ M_0 l^2 }{ 20 E I(x) } \alpha \lambda ^2 \left( 1+\nu \right) \left( 1-\alpha \xi \right) ^2 \left( 2-3\alpha \xi \right) \end{aligned}$$

A4 $$\begin{aligned}  &   w_{HO}(x) = \frac{ M_0 l^2 }{ 1120 E I(x) } \alpha \lambda ^2 \left( 1-\alpha \xi \right) ^2 \xi \left[ 56 \nu \left( 2+\alpha \xi \right) + \alpha ^2 \lambda ^2 \left( 4-11\alpha \xi \right) \right] \end{aligned}$$

Note that if the tapered cantilever is loaded by the end moment $M_0$, the shear dependent term (A3) does not vanish $w_{shear}(x) \ne 0$: although the shear force is zero, the local shear stress is nonzero in Eq. (22) because of $I (x) \ne 0$ and $M^*_{,x} (x) \ne 0$.

**A.1.1 Tip deflection** Evaluating Eqs. (A1)–(A4) at $x=l$, the vertical deflection reads

A5 $$\begin{aligned}  &   w (x=l) = w_{BE}(l) + w_{\textrm{shear}}(l) + w_{\textrm{HO}}(l) \end{aligned}$$

A6 $$\begin{aligned}  &   w_{BE}(l) = - \frac{ M_0 l^2 }{ 2 E I(l) } \left( 1-\alpha \right) ^2 \end{aligned}$$

A7 $$\begin{aligned}  &   w_{shear}(l) = \frac{ M_0 l^2 }{ 20 E I(l) } \alpha \lambda ^2 \left( 1+\nu \right) \left( 1-\alpha \right) ^2 \left( 2-3\alpha \right) \end{aligned}$$

A8 $$\begin{aligned}  &   w_{HO}(l) = \frac{ M_0 l^2 }{ 1120 E I(l) } \alpha ^3 \lambda ^2 \left( 1-\alpha \right) ^2 \left[ 56 \nu \left( 2+\alpha \right) + \alpha ^2 \lambda ^2 \left( 4-11\alpha \right) \right] \end{aligned}$$

### A.2 Analytical deflection curve of a tapered cantilever with tip load

Considering the bending moment and the shear force distributions $M(x) = -F_1 (l-x)$ and $Q(x) = F_0$, one finds for the deflection curve

A9 $$\begin{aligned}  &   w (x) = w_{BE}(x) + w_{\textrm{shear}}(x) + w_{\textrm{HO}}(x) \end{aligned}$$

A10 $$\begin{aligned}  &   \quad w_{BE}(x) = - \frac{ F_0 l^3 }{ 2 E I(x) \alpha ^3 } \left( 1-\alpha \xi \right) ^2 \left[ \alpha \xi \left( 2-\alpha \xi - \alpha ^2 \xi \right) \right. \left. + 2 \left( 1-\alpha \xi \right) \log \left( 1-\alpha \xi \right) \right] \end{aligned}$$

A11 $$\begin{aligned}  &   w_{shear}(x) = \frac{ F_0 l^3 }{ 20 E I(x) \alpha } \left( 1-\alpha \xi \right) ^2 \lambda ^2 \left( 1+\nu \right) \nonumber \\  &   \quad \qquad \qquad \qquad \left[ \alpha \xi \left( -2-2\alpha \xi +2\xi + 3 \alpha ^2\xi \right) - 6 \left( 1-\alpha \xi \right) \log \left( 1-\alpha \xi \right) \right] \end{aligned}$$

A12 $$\begin{aligned}  &   w_{HO}(x) = - \frac{ F_0 l^3 }{ 1120 E I(x) \alpha } \left( 1-\alpha \xi \right) ^2 \lambda ^2 \nonumber \\  &   \quad \qquad \qquad \qquad \left[ \alpha \xi \left( 56\nu (-6+\alpha ^2 \xi + 2\alpha +3\alpha \xi ) +\alpha ^2\lambda ^2 (14-11\alpha ^2\xi +4\alpha -7\alpha \xi ) \right) \right. \nonumber \\  &   \qquad \qquad \qquad \left. + 6 \left( 5\alpha ^2 \lambda ^2-56\nu \right) \left( 1-\alpha \xi \right) \log \left( 1-\alpha \xi \right) \right] \end{aligned}$$

**A.2.1 Tip deflection** Evaluating Eqs.(A9)–(A12) at $x=l$, the vertical deflection reads

A13 $$\begin{aligned}  &   w (x=l) = w_{BE}(l) + w_{\textrm{shear}}(l) + w_{\textrm{HO}}(l) \end{aligned}$$

A14 $$\begin{aligned}  &   w_{BE}(l) = -\frac{ F_0 l^3 }{ 2 E I(l) \alpha ^3 } \left( 1-\alpha \right) ^3 \left[ \alpha \left( 2+\alpha \right) + 2 \log \left( 1-\alpha \right) \right] \end{aligned}$$

A15 $$\begin{aligned}  &   w_{shear}(l) = -\frac{ F_0 l^3 \lambda ^2 }{ 20 E I(l) \alpha } \left( 1-\alpha \right) ^3 \left( 1+\nu \right) \left[ \alpha \left( 2+3\alpha \right) + 6 \log \left( 1-\alpha \right) \right] \end{aligned}$$

A16 $$\begin{aligned}  &   w_{HO}(l) = -\frac{ F_0 l^3 \lambda ^2 }{ 1120 E I(l) \alpha } \left( 1-\alpha \right) ^3 \left[ 6 \left( 5\alpha ^2 \lambda ^2-56\nu \right) \log \left( 1-\alpha \right) \right. \nonumber \\  &   \qquad \qquad \qquad \left. + \alpha \left( 14\alpha ^2\lambda ^2 +11\alpha ^3\lambda ^2-336\nu -56\alpha \nu \right) \right] \end{aligned}$$

### A.3 Analytical deflection curve and axial deflection of a tapered cantilever with distributed load

Considering the bending moment and the shear force distributions $M(x) = -q_0 (l-x)^2/2$ and $Q(x) = q_0 (l-x)$ caused by the distributed load $q_0$ over the lower surface, the deflection curve obtained by Castigliano’s method reads

A17 $$\begin{aligned}  &   w (x) = w_{BE}(x) + w_{\textrm{shear}}(x) + w_{\textrm{HO}}(x) \end{aligned}$$

A18 $$\begin{aligned}  &   w_{BE}(x) = \frac{ q_0 l^4 }{ 4 E I(x) \alpha ^4 } \left( 1-\alpha \xi \right) ^2 \left[ \alpha \xi \left( 6+2\alpha ^2 \xi + \alpha ^3 \xi -\alpha (4+5\xi ) \right) \right. \nonumber \\  &   \quad \quad \qquad \qquad \left. + 2 \left( 3+\alpha ^2 \xi (2+\xi )-2\alpha (1+2\xi ) \right) \log \left( 1-\alpha \xi \right) \right] \end{aligned}$$

A19 $$\begin{aligned}  &   w_{shear}(x) = \frac{ q_0 l^4 \lambda ^2 }{ 40 E I(x) \alpha ^2 } \left( 1-\alpha \xi \right) ^2 \left( 1+\nu \right) \left[ \alpha \xi \left( 14-2\alpha ^2(1- \xi )+3\alpha ^2\xi \right. \right. \nonumber \\  &   \quad \quad \qquad \qquad \left. \left. -\alpha (4+13\xi ) \right) + 2 \left( 1-\alpha \xi \right) \left( 7-6\alpha -\alpha \xi \right) \log \left( 1-\alpha \xi \right) \right] \end{aligned}$$

A20 $$\begin{aligned}  &   w_{HO}(x) = \frac{ q_0 l^4 \lambda ^2 }{ 2240 E I(x) \alpha ^2} \left( 1-\alpha \xi \right) ^2 \left[ \alpha \xi \left( -56\nu \left( 14+\alpha ^3 \xi + \alpha ^2(2+6\xi ) \right. \right. \right. \nonumber \\  &   \quad \quad \qquad \qquad \left. \left. -\alpha (12+11\xi )\right) +\alpha ^2\lambda ^2 \left( 42+11\alpha ^3\xi -7 \alpha (4+5\xi )+2\alpha ^2(-2+7\xi ) \right) \right) \nonumber \\  &   \quad \quad \qquad \qquad \left. +2\left( 1-\alpha \xi \right) \left( 56\nu (7-\alpha (6+\xi )) +3\alpha ^2\lambda ^2(11-\alpha (10+\xi )) \right) \log \left( 1-\alpha \xi \right) \right] \end{aligned}$$

Note that the distributed load $q_0$ causes the transverse normal stress $\sigma _{zz} \ne 0$, see Figs. 7c and 8c. The axial deflection increases linearly

A21 $$\begin{aligned}  &   u (x) = u_{\textrm{shear}}(x) + w_{\textrm{HO}}(x) \end{aligned}$$

A22 $$\begin{aligned}  &   u_{shear}(x) = 0 \end{aligned}$$

A23 $$\begin{aligned}  &   u_{HO}(x) = \frac{ q_0 l^4 \lambda ^2 }{ 288 E I(x) } \left( \alpha ^2 \lambda ^2 -12\nu \right) \xi \left( 1-\alpha \xi \right) ^3 \end{aligned}$$

Note that $I(x) = 2b c_0^3 (1-\alpha \xi )^3/3$ holds.

**A.3.1 Tip deflections** Evaluating Eqs. (A17)–(A21) at $x=l$, the vertical and the axial tip deflections read

A24 $$\begin{aligned}  &   w (x=l) = w_{BE}(l) + w_{\textrm{shear}}(l) + w_{\textrm{HO}}(l) \end{aligned}$$

A25 $$\begin{aligned}  &   w_{BE}(l) = \frac{ q_0 l^4 }{ 4 E I(l) \alpha ^4 } \left( 1-\alpha \right) ^2 \left[ \alpha \left( 6-9\alpha -2\alpha ^2 + \alpha ^3 \right) \right. \left. + 6 \left( 1-\alpha \right) ^2 \log \left( 1-\alpha \right) \right] \end{aligned}$$

A26 $$\begin{aligned}  &   w_{shear}(l) = \frac{ q_0 l^4 \lambda ^2 }{ 40 E I(l) \alpha ^2 } \left( 1-\alpha \right) ^3 \left( 1+\nu \right) \left[ \alpha \left( 14-3\alpha - 3\alpha ^2 \right) \right. \left. + 14 \left( 1-\alpha \right) \log \left( 1-\alpha \right) \right] \end{aligned}$$

A27 $$\begin{aligned}  &   w_{HO}(l) = \frac{ q_0 l^4 \lambda ^2 \left( 1-\alpha \right) ^3 }{ 2240 E I(l) \alpha ^2 } \left[ 2\left( 1-\alpha \right) \left( 33\alpha ^2\lambda ^2-392\nu \right) \log \left( 1-\alpha \right) \right. \nonumber \\  &   \quad \qquad \qquad \qquad \left. -\alpha \left( 21 \alpha ^3 \lambda ^2+11\alpha ^4\lambda ^2+784\nu -504\alpha \nu -14\alpha ^2 (3\lambda ^2+4\nu ) \right) \right] \nonumber \\ \end{aligned}$$

and

A28 $$\begin{aligned} u (l) = \frac{ q_0 l^4 \lambda ^2 }{ 288 E I(l) } \left( \alpha ^2 \lambda ^2 -12\nu \right) \left( 1-\alpha \right) ^3 \end{aligned}$$
